# Association of salivary proteins with dental caries in children with mixed dentition: a systematic review

**DOI:** 10.1007/s40368-024-00994-4

**Published:** 2025-01-18

**Authors:** M. Raja, H. Nazzal, F. S. Cyprian, M. Matoug- Elwerfelli, M. Duggal

**Affiliations:** 1 Qatar University Health, College of Dental Medicine, Qatar University, Doha, Qatar; 2 Paediatric Dentistry, Hamad Medical Corporation, Doha, Qatar; 3 Qatar University Health, College of Medicine, Qatar University, Doha, Qatar

**Keywords:** Dental caries, Children, Mixed dentition, Salivary proteins

## Abstract

**Purpose:**

To review the current evidence on the association between salivary protein profile and dental caries in children during mixed dentition stage.

**Methods:**

This systematic review followed the PRISMA 2020 guidelines. Searches were run in PubMed, Scopus and Embase along with gray literature. The searches were limited to studies on humans, published from inception to February 2024. Observational studies exploring correlations between salivary proteins and caries in children during mixed dentition (6–13 years) were included. The methodological quality of included studies was analyzed independently by two reviewers using the Joanna Briggs checklist and Newcastle–Ottawa scale, respectively followed by a qualitative synthesis.

**Results:**

A total of 17 primary studies were included. The studies recruited 1,330 subjects including 612 caries-active and 505 caries-free subjects. The total protein content was higher in caries-active subjects. Among the glycoproteins, IgA and MUC5B were higher in caries-free subjects while the levels of MUC7 were lower in the caries-free group. Antimicrobial peptides and proteinase-3 levels were also higher in caries-free subjects. Contradictory findings were reported for the association of α- amylase and carbonic anhydrase with caries status. The included studies were categorized as good quality (*n* = 4), fair quality (*n* = 12) and poor quality (*n* = 1).

**Conclusion:**

Based on fair-quality studies included in this review and within its limitations, the findings revealed that salivary proteins may be associated with susceptibility to dental caries in mixed dentition. Total salivary proteins are upregulated in caries-active subjects while salivary IgA, antimicrobial peptides and proteinase-3 are increased in caries-free subjects.

*Systematic review registration*: The study protocol was registered in PROSPERO (registration number CRD42024517374).

**Supplementary Information:**

The online version contains supplementary material available at 10.1007/s40368-024-00994-4.

## Introduction

Dental caries is the most common non-communicable chronic disease worldwide and affects individuals of all age-groups and is the most common preventable childhood disease (Selwitz et al. 2007). Although the prevalence of dental caries has decreased in developed countries in recent years, the global burden of caries continues to remain significant. A recent systematic review reported 2.3 billion cases (95% UI, 2.1 to 2.5 billion) of untreated caries in permanent teeth (Bernabe et al. 2020). Caries is a complex and multifactorial disease affecting the mineralized dental tissues which is initiated within the bacterial biofilm (dental plaque) which covers teeth and oral soft tissues. Oral bacteria ferment dietary carbohydrates leading to the production of acidic by-products which demineralize dental hard tissues (Schwendicke, Frencken and Innes 2018). Caries involves microbiological shifts within the complex biofilm and is affected by salivary flow, composition, exposure to fluoride, consumption of dietary sugars, and preventive measures including oral hygiene (Selwitz et al. 2007).

Risk factors for dental caries include poor oral hygiene, frequent exposure to dietary sugars, high numbers of cariogenic bacteria, insufficient fluoride exposure, inadequate salivary flow, inappropriate methods of feeding infants, previous caries experience, and poverty (Petersen et al. 2005; Selwitz et al. 2007). Over the years, a growing interest has been observed in exploring the value of salivary proteins as potential biomarkers of dental caries. Human saliva contains more than 2000 distinct types of salivary proteins and peptides. These include antimicrobial peptides, such as cathelicidin, histatins, defensins, statherins; glycoproteins, such as mucins, proline-rich proteins, immunoglobulin A, agglutinin, lactoferrin, cystatins, and lysozyme (Tao et al. 2005; Hemadi et al. 2017). In addition, enzymes such as carbonic anhydrase offer protection against caries due to their buffering capacity (Kivelä et al. 2003; Abdelaal et al. 2023). Given that saliva contains a plethora of protective factors against dental caries, a large number of research studies have focused on protein analysis of saliva in an attempt to identify potential biomarkers associated with either protection against caries as well as those indicating an increased risk (Ayad et al. 2000; Tao et al. 2005; Zakharv et al. 2007; Rudney et al. 2009; Zehetbauer et al. 2009; Sun et al. 2016; Ao et al. 2017). Several systematic reviews have also been published on the potential role of salivary proteins as biomarkers for dental caries (Martins et al. 2013; Umashankar and Ramani 2021; Ahmad et al. 2022). However, the existing studies do not provide adequate evidence to support the development of a predictable and reliable model of caries-risk assessment based on salivary proteins.

The mixed dentition period is unique in several ways. It represents a transition from primary to permanent dentition, but also the children may share common dietary patterns, oral hygiene habits, and profile of oral microbiome (Shi et al. 2016; Mason et al. 2018). It is also likely that salivary protein expression among children with mixed dentition may also be different to adults (Sivakumar et al. 2009).Therefore, the aim of the current review was to systematically review and analyze all the available evidence on the potential association between salivary proteins and dental caries in children during the mixed dentition stage.

## Methods

### Protocol and registration

The systematic review protocol was registered at the National Institute for Health Research (PROSPERO), International Prospective Register of Systematic Reviews (https://www.crd.york.ac.uk/prospero/display_record.php?RecordID=517374 (PROSPERO 2024, registration number CRD42024517374). This systematic review is reported as per the Preferred Reporting Items for Systematic Reviews and Meta-Analyses (PRISMA) 2020 statement (https://www.prismastatement.org). The checklists for PRISMA guideline are included in the appendix (Table I and Table II).

### Focused question

In children with mixed dentition, is there an association between salivary protein profile and occurrence of dental caries?

### PECOS framework and eligibility criteria

The study *population* was children during mixed dentition stage (6–13 years). Exclusion criteria were studies performed on children with known systemic disease requiring regular medical care; children with physical or mental disabilities; children with developmental anomalies of the oral and maxillofacial region and children taking medications. The *exposure* was salivary protein profile in children with dental caries while the *comparator* was salivary protein profile in caries-free children. The main *outcome* was difference in salivary protein levels and association with dental caries. Original human research having an observational methodological study design (cohort, case–control, and analytical cross-sectional studies) was included. Animal studies, in vitro studies, reviews, editorials, commentaries, abstracts, research protocols, and articles published in languages other than English were excluded.

### Information sources and search strategy

Three electronic databases, namely PubMed, Scopus, and Embase, were used to search for relevant studies. In addition, Google Scholar was searched for any eligible studies. A supplementary search in the gray literature was undertaken using Open Grey (https:// www.opengrey.eu). Moreover, the reference list from retrieved full-text articles was examined and published review articles were searched manually to identify additional studies.

A comprehensive systematic search strategy was used with appropriate syntax for individual databases. A combination of key words and index terms was used by integrating Boolean operators to create meaningful search strings.

The following search strategy was used in PubMed and adapted for other databases:

(((((((((((((((((((((("saliva proteins") OR ("saliva peptides")) OR ("saliva proteome")) OR ("salivary proteins")) OR ("salivary peptides")) OR ("Mucin-5B"[Mesh])) OR ("Salivary Proline-Rich Proteins"[Mesh])) OR ("Salivary alpha-Amylases"[Mesh])) OR ("Histatins"[Mesh])) OR ("Salivary Cystatins"[Mesh])) OR ("salivary protein biomarkers")) OR ("salivary proteomic profile")) OR ("total salivary proteins")) OR ("salivary mucins")) OR ("salivary IgA")) OR ("salivary statherin")) OR ("salivary defensins")) OR (salivary cathelicidins)) OR (salivary human lysozyme)) OR ("salivary lactoferrin")) OR ("salivary glycoproteins")) OR (salivary proteinase 3)) **AND** ((((((((("Dental Caries"[Mesh]) OR ("Dental Caries Susceptibility"[Mesh])) OR ("caries")) OR ("carious lesion")) OR ("tooth demineralisation")) OR ("dental decay")) OR ("tooth cavities")) OR ("white spot lesions")) OR ("tooth decay")).

The searches were carried out on 16 February 2024. Details of search terms used for individual databases are provided in the supplementary data file (Table III).

### Study selection process

All the identified records were imported into reference management software (desktop version of EndNote®, version X20; Clarivate Analytics) and duplicates were removed. Title and abstract screening of the studies was done independently by two investigators (M.R and M.M.E) as recommended (Rosenthal, 1991), using Rayyan Systematic Review Screening Software (https://www.rayyan.ai) based on eligibility criteria.

Full texts of potentially eligible studies were retrieved and evaluated independently by two reviewers (M.R and M.M.E) using the same method. Any disagreements in screening were discussed and resolved by a third reviewer (H.N). Articles that did not meet any one or more of the inclusion criteria were excluded. A log of excluded studies along with the justification for exclusion was maintained.

### Data collection process

Data extraction was performed by two reviewers (M.R and M.M.E) independently and comparisons were done to evaluate accuracy of data. Any disagreement was resolved through discussion between the two reviewers. The key data extracted from selected literature were: a) study Information (author, year and country of publication); (b) study design; (c) age of subjects; (d) sample size; (e) gender; (f) sample size; (g) study groups; (h) caries index; (I) saliva sample; (j) salivary proteins quantification method; (k) type of salivary proteins assessed; (l) salivary proteins levels/expression; (m) statistical significance; (*n*) main findings; and (o) conclusion. The data were recorded in a standardized Microsoft Excel sheet. The corresponding authors of studies with missing or poorly reported data were contacted. However, no responses were received and only published data were used.

### Methodological quality (risk of bias) assessment

The quality assessment was conducted by two reviewers (M.R and M.M.E) independently and any differences were resolved through discussion. The methodological quality of individual studies was assessed using appropriate tools according to the study design.

The Joanna Briggs Institute (JBI) critical appraisal tool was used for analytical cross-sectional studies (JBI 2020). This tool assessed studies on eight criteria each of which was graded as “Yes”, “No”, “Unclear” or “Not applicable to address the possibility of bias in its design, conduct, and analysis. Quality assessment was calculated by dividing the frequency of “yes” answers above the total number of questions. The studies were characterized as having a high risk of bias (poor quality) for less than 49% items as “yes”; moderate (fair quality) between 50 and 69% items as “yes”; and low risk of bias (good quality) when more than 70% of the items answered as “yes”^.^(dos Anjos et al. 2023; Normando et al. 2023).

For case–control and cohort studies, the methodological quality assessment was carried out using the Newcastle–Ottawa Scale (NOS) (0–9 asterisks) (Wells et al. 2012). NOS is a three-dimensional appraisal tool that included selected population (0–4 stars), comparability of the study groups by controlling for relevant factors (0–2 stars), and exposure /outcome domain (0–3 stars). An overall estimation of quality was undertaken using the following thresholds: Good quality,7 stars or more; Fair quality, 4–6 stars; or poor quality, 0–3 stars (Stang 2010).

### Synthesis methods

Qualitative and descriptive data synthesis was performed for all the included studies. Quantitative synthesis could not be performed due to marked heterogeneity in the included studies in relation to methodology and inconsistencies in the reported outcomes which precluded a meta-analysis. Therefore, only descriptive and narrative synthesis of the results was possible.

## Results

### Study literature search and selection.

**Fig. 1:**
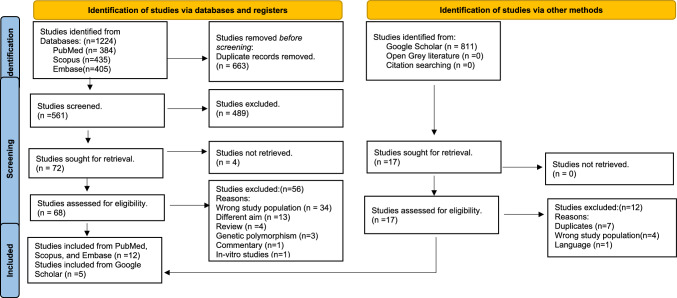
PRISMA flow diagram of the processes leading to 17 studies included for review (Page et al. 2021)

A total of 1,224 studies were initially retrieved from the three electronic databases. These were reduced to 561 after removal of 663 duplicates. Following a double title/abstract screening of 561 studies, 489 articles were excluded due to non-conformity with the eligibility criteria. The remaining 72 records were identified for full-text screening. Full texts of four articles could not be retrieved. Through a meticulous full-text screening process, a total of 68 studies were assessed. Subsequently, 56 articles were excluded due to their failure to meet the eligibility criteria. The reasons for exclusion are provided in the supplementary file (Table IV). Finally, 12 studies were identified for inclusion from PubMed, Scopus and Embase. A parallel search on Google Scholar identified 811 studies of which 17 were assessed for eligibility. Following exclusion of 12 studies, 5 were found to be eligible for inclusion in the review after full-text screening. Combined search, screening, and selection process of studies from PubMed, Scopus, Embase, and Google Scholar identified 17 primary studies for inclusion in the review as depicted in the PRISMA Flow chart (Fig. 1). No additional records were retrieved from open gray literature.

### Primary characteristics of individual studies

Of the 17 primary studies included in this systematic review, 14 studies (82.35%) employed an analytical cross-sectional study design. Other study designs included 2 case–control studies (11.76%) and one prospective cohort study (5.88%). The included studies were published between 2011 and 2022. The main characteristics of the included studies in the qualitative synthesis are summarized in Table 1. A total of 1,330 subjects were recruited of which 537 were males, and 559 were females. Five studies did not report male-to-female distribution (Damle and Doifode 2011; Ranadheer et al. 2011; Priya et al. 2013; Picco et al. 2017; Vasudevan et al. 2022). Most studies were conducted in India (*n* = 6) (Damle and Doifode 2011; Ranadheer et al. 2011; Priya et al. 2013; Pyati et al. 2018; Ahmad et al. 2021; Vasudevan et al. 2022), followed by China (*n* = 3) (Yang et al. 2015; Wang et al. 2018; Chen et al. 2020), Brazil (*n* = 1) (Picco et al. 2017), Colombia (*n* = 1) (Angarita-Díaz et al. 2021), Egypt (*n* = 1) (WM and Youssef 2016), Indonesia (*n* = 1) (Soesilawati et al. 2019) Romania (*n* = 1) (Monea, Vlad and Stoica 2018), Saudi Arabia (*n* = 1) (Murugeshappa et al. 2018), Serbia (*n* = 1) (Stojković et al. 2020), and Thailand (*n* = 1) (Angwaravong et al. 2015).

**Table 1:** Primary characteristics of the included studies

Author/Country	Study design	Participant Age (Years)	Participant Gender (Male\Female)	Sample size (*n*) & groups	Diagnostic criteria	Saliva Sample collection	Salivary proteins assessed	Salivary proteins quantification method(s)
Ahmad et al. 2021 India	Analytical cross-sectional	8–12	48 males 52 females	Caries-active (*n* = 50) Caries-free (*n* = 50)	Caries-active: (DMFT/deft ≥ 5) Caries-free: (DMFT/or deft = 0)	2-3 mL unstimulated saliva (09:00 -10:00)	Salivary IgA α- amylase	Sandwich enzyme immunoassay
Angarita-Díaz et al. 2021 Colombia	Analytical cross-sectional	6–12	14 males 19 females	Caries-active (*n* = 21) Caries-free (*n* = 12)	ICDAS	2 mL unstimulated saliva (08:00 -11:00)	Salivary IgA Cathelicidin LL-37 Statherin Fibronectin	ELISA
Angwaravong et al. 2015 Thailand	Analytical cross-sectional	9–11	High caries-risk 16 males;14 females Low caries-risk 16 males;14 females	High caries-risk (*n* = 30) Low caries-risk (*n* = 30)	Modified WHO diagnostic criteria HCR group: (≥ 1carious tooth) LCR group: (< 1)	Unstimulated saliva (09:00 -11:00)	Salivary mucins (MUC5B; MUC7)	ELISA
Chen et al. 2020 China	Analytical cross-sectional	6–8	Equal number of males and females in both groups	Caries-active (*n* = 40) Caries-free (*n* = 40)	Caries -active: (dmfs > 8) Caries-free: (dmfs = 0)	Unstimulated saliva Morning	Differentially expressed salivary proteins: (lactoferrin, mucin, matrix metalloproteinase, cystatin, immunoglobulin, peptides, protein S100, & proline-rich proteins)	Bradford assay SDS-PAGE electrophoresis iTRAQ-coupled LC–MS/MS
Damle and Doifode., 2011 India	Analytical cross-sectional	8–10	NR	Caries-active (*n* = 15) Caries-free (*n* = 15)	Caries-active: (dfs ≥ 10) Caries-free: (dfs = 0)	2 mL Unstimulated saliva Collection time NR	Salivary IgA	Radial immunodiffusion
EK and Youssef., 2016 Egypt	Analytical cross-sectional	6–12	Equal number of males and females in both groups	Caries-active (*n* = 40) Caries-free (*n* = 40)	WHO diagnostic criteria	Unstimulated Saliva Collection time NR	Total salivary proteins α-defensin	Biuret method ELISA
Monea et al. 2018 Romania	Analytical cross-sectional	9–12	77 males 65 females	Caries-active (*n* = 97) Caries-free (*n* = 45)	Visual detection method	10 mL Unstimulated saliva Morning	Salivary α-amylase	Spectrophotometer
Murugeshappa et al. 2018 Saudi Arabia	Analytical cross-sectional	7–12	Caries-active 8 males; 27 females Caries-free 16 males;19 females	Caries-active (*n* = 35) Caries-free (*n* = 35)	Caries-active: (DMFT ≥ 5) Caries-free: (DMFT = 0)	Unstimulated saliva Collection time NR	Total salivary proteins Salivary IgA	Bradford method ELISA
Picco et al. 2017 Brazil	Analytical cross-sectional	7–9	NR	Caries-active (*n* = 37) Caries-free (*n* = 37)	DMFT	I mL Stimulated saliva Collection time NR	Carbonic anhydrase VI concentration	ELISA
Priya et al. 2013 India	Analytical cross-sectional	7–12	NR	Caries-active (*n* = 15) Caries-free (*n* = 15)	Caries-active: (DMFT/deft score ≥ 5) Caries-free: (DMFT/deft score 0)	2–3 mL Unstimulated saliva Collection time NR	Salivary IgA	ELISA
Pyati et al. 2018 India	Analytical cross-sectional	6–12	Caries-active 29 males;21 females Caries-free 28 males;22 females	Caries-active (*n* = 50) Caries-free (*n* = 50)	Caries-active: (DMFS/dfs ≥ 5) Caries-free: (DMFS/dfs = 0)	Unstimulated saliva (10:00 -11:30)	Total salivary protein	Biuret method
Ranadheer et al. 2011 India	Analytical cross-sectional	8–12	NR	Caries-active (*n* = 20) Caries-free (*n* = 20)	Caries-active: (DMFT ≥ 3) Caries-free: (DMFT = 0)	Unstimulated saliva Collection time NR	Salivary IgA	ELISA
Soesilawati et al. 2019 Indonesia	Case–control	6–9	Caries-active 11 males;19 females Low caries-active 11 males;19 females	Caries-active (*n* = 30) Low caries-active (*n* = 30)	Caries-active: (deft ≥ 3) Low caries-active: (deft < 3)	Stimulated saliva Morning (10:00 -12:00)	Salivary IgA	ELISA
Stojković et al. 2020 Serbia	Prospective cohort	11–13	99 males 114 females	213 children	Caries-active: (DMFT ≥ 1) Caries-free: (DMFT = 0)	2 mL Unstimulated saliva Morning	HNP-1 hBD-2 LL-37 (Antimicrobial peptides)	ELISA
Vasudevan et al. 2022 India	Case–control	6–12	NR	Caries- active (*n* = 30) Caries-free (*n* = 30)	Caries-active: (DMFT)/dmft > 5) Caries-free: (DMFT/dmft = 0)	6 mL Stimulated saliva (10:00 -11:30)	CA VI iso-enzyme Total salivary protein	ELISA
Wang et al. 2018 China	Analytical cross-sectional	10–12	16 males 14 females	HDC( *n* = 10) LDC (*n* = 10) NDC (*n* = 10)	HDC group: (DMFT/dmft 5–10) LDC group: (DMFT/dmft 1 to 4) NDC group: (DMFT = 0)	3 mL Unstimulated saliva (09:00 -11:00)	Differentially expressed salivary proteins: (mucin 7, mucin 5B, histatin 1, cystatin S & cystatin SN, basic salivary proline-rich protein 2)	iTRAQ‑based mass spectrometry MRM mass spectrometry
Yang et al. 2015 China	Analytical cross-sectional	6	68 males 60 females	HDC (*n* = 33) LDC (*n* = 49) NDC (*n* = 46)	HDC group: DMFT/deft = (5–15) LDC group: DMFT/deft = (1–4) NDC group: (DMFT/deft = (0)	5 mL Unstimulated saliva (08:00 -10:00)	Proteinase 3 (PR3)	ELISA

CA VI = Carbonic Anhydrase VI; dmfs = Index of decayed, missing due to caries, or filled tooth surfaces; DMFT = Decayed, Missing, Filled Teeth; ELISA = Enzyme-linked immunosorbent assay; HBD-2 = Human beta-defensin-2; HDC = High dental caries; HNP-1 = Human Neutrophil Peptide-1; ICDAS: International Caries Detection Assessment System; iTRAQ = Isobaric tags for relative and absolute quantitation; LC–MS: Liquid chromatography–mass spectrometry; LDC = Low dental caries; MRM = Multiple reaction monitoring; NDC = No dental caries; NR = Not reported; SDS-PAGE: Sodium dodecyl sulfate–polyacrylamide gel electrophoresis; sIgA = salivary immunoglobulin A

The included studies investigated a variety of salivary proteins for their potential association with dental caries in children during mixed dentition (6–13 years). Most of the studies used DMFT/deft caries index (*n* = 13) (Damle and Doifode 2011; Ranadheer et al. 2011; Priya et al. 2013; Yang et al. 2015; Picco et al. 2017; Murugeshappa et al. 2018; Pyati et al. 2018; Wang et al. 2018; Soesilawati et al. 2019; Chen et al. 2020; Stojković et al. 2020; Ahmad et al. 2021; Vasudevan et al. 2022), except for 3 studies that employed the ICDAS (*n* = 1)(Angarita-Díaz et al. 2021), visual detection method (*n* = 1) (Monea, Vlad and Stoica 2018), and modified WHO diagnostic criteria (*n* = 1) (Angwaravong et al. 2015). Although most studies used DMFT/deft caries index, the cut-off values of caries-free/caries-prone patients varied among studies. There were 612 individuals with dental caries and 505 subjects in the control group across all the studies. Five studies considered (DMFT/deft ≥ 5) as caries-active group (Priya et al. 2013; Murugeshappa et al. 2018; Pyati et al. 2018; Ahmad et al. 2021; Vasudevan et al. 2022), two studies considered (DMFT/deft ≥ 3) as caries-active group (Ranadheer et al. 2011; Soesilawati et al. 2019), one study considered (dmfs > 8) as caries-active group (Chen et al. 2020), whereas one study considered (dfs ≥ 10) as caries-active group (Damle and Doifode 2011). Two studies considered (DMFT/dmft = 5–10 & 5–15) as high dental caries group, and (DMFT/dmft = 1–4) as low dental caries group (Yang et al. 2015; Wang et al. 2018), while two studies did not report the DMFT/deft scores (WM and Youssef 2016; Picco et al. 2017). On the other hand, studies considered DMFT /or deft = 0 as caries-free group (*n* = 12) (Damle and Doifode 2011; Ranadheer et al. 2011; Priya et al. 2013; Angwaravong et al. 2015; Yang et al. 2015; Murugeshappa et al. 2018; Pyati et al. 2018; Wang et al. 2018; Chen et al. 2020; Stojković et al. 2020; Ahmad et al. 2021; Vasudevan et al. 2022). Only one study considered deft < 3 as low-caries group (Soesilawati et al. 2019).

All included studies collected unstimulated saliva, except for three studies that evaluated stimulated saliva (Picco et al. 2017; Soesilawati et al. 2019; Vasudevan et al. 2022). Most studies performed saliva collection in the morning; however, six studies did not report at what time of day the saliva sample collections were carried out (Damle and Doifode 2011; Ranadheer et al. 2011; Priya et al. 2013; WM and Youssef 2016; Picco et al. 2017; Murugeshappa et al. 2018). Of the 17 studies, only two studies evaluated differentially expressed salivary proteins between caries-free and caries affected individuals (Wang et al. 2018; Chen et al. 2020) while the remaining 15 studies compared salivary levels of specific proteins among caries-free and caries-active groups.

In regard to the methods for protein analysis, enzyme-linked immunosorbent assay (ELISA) was used for quantification of salivary proteins in most studies (Ranadheer et al. 2011; Priya et al. 2013; Angwaravong et al. 2015; Yang et al. 2015; WM and Youssef 2016; Picco et al. 2017; Murugeshappa et al. 2018; Soesilawati et al. 2019; Stojković et al. 2020; Angarita-Díaz et al. 2021; Vasudevan et al. 2022). Total protein content of saliva was investigated by Biuret method (WM and Youssef 2016; Pyati et al. 2018), and Bradford analysis (Murugeshappa et al. 2018; Chen et al. 2020). Other methods used were spectrophotometer (Monea, Vlad and Stoica 2018), radial immunodiffusion (Damle and Doifode 2011), and two-site sandwich enzyme immunoassay (Ahmad et al. 2021). Two studies carried out a comprehensive analysis of salivary proteome with a focus on evaluating differentially expressed proteins reported in caries-free and caries-active children (Wang et al. 2018; Chen et al. 2020) Chen et al (2020) used SDS-PAGE electrophoresis, and iTRAQ-coupled LC–MS/MS for salivary protein quantification (Chen et al. 2020), while Wang et al (2018) also used iTRAQ-based mass spectrometry for quantitative proteomic analysis (Wang et al. 2018).

### Association between salivary proteins and dental caries

The findings of the included studies based on differences in salivary protein levels between caries-active and caries-free groups are depicted in Table 2. The key findings related to different salivary proteins are summarized below.

**Table 2:** Association of salivary proteins with dental caries in children with mixed dentition in the included studies

Study	Salivary proteins in caries-active subjects (Mean ± SD)	Salivary proteins in caries-free subjects (Mean ± SD)	Significance ( *p*-value)	Main findings	Conclusions
Ahmad et al. 2021	sIgA (μg/ml) = 2.98 ± 1.66 α amylase (U/ml) = 68.42 ± 26.28	sIgA (μg/ml) = 5.62 ± 1.77 α amylase (U/ml) = 83.53 ± 27.61	*p* = 0.001 *p* = 0.014	Mean salivary IgA and amylase levels in caries-free subject increased significantly	Higher levels of sIgA and α amylase identified in caries-free children
Angarita-Díaz et al. 2021	IgA = Median: 37,776.42 IQR: 33,383.9–44,128.5 LL-37 = Median:46.3 IQR:40.1011–67.7 Statherin = Median:93,199.1 IQR:87,737.9–94,587.9 Fibronectin = Median:16.7 IQR:11.9–41.1	IgA = Median:48,250.0 IQR: 31,461.9–67,418.8 LL-37 = Median:56.1 IQR: 43.6–116.2 Statherin = Median:94,734.6 IQR: 92,934.6–95,113.7 Fibronectin = Median:20.43 IQR:13.8– 34.2	*p* = 0.12 *p* = 0.56 *p* = 0.03 *p* = 0.7	IgA, cathelicidin LL-37, statherins, & fibronectin higher in the caries-free children	A significantly higher statherin concentration was detected in caries-free subjects For other proteins differences between groups not significant
Angwaravong et al. 2015	MUC5B (HCR group) = Median; IQR MUC7 (HCR group) = Median; IQR Numerical values not reported	MUC5B (LCR group) = Median; IQR MUC7 (LCR group) = Median; IQR Numerical values not reported	*p* = 0.01 *p* = 0.04	Significantly increased MUC5B & decreased MUC7 levels in subjects with low-caries-risk group	Changes in oral environment in mixed dentition may affect the secretion of saliva
Chen et al. 2020	Differentially expressed proteins	Differentially expressed proteins	P < 0.05 Ratio-fold change > 1.2	9135 unique peptides & 1662 proteins identified 258 proteins differentially expressed between the caries-free and caries-active group	258 differentially expressed proteins could be associated with caries status (Lactoferrin, mucin-7, matrix metalloproteinase-9, cystatin, immunoglobulin peptides, protein S100-A9 & proline-rich protein associated with caries)
Damle and Doifode., 2011	sIgA (mg/dl) = 8.98 ± 1.56	IgA level(mg/dl) = 10.74 ± 1.52	*p* = 0.012	Whole sIgA levels were significantly higher in caries-free subjects	sIgA may have a role in immunological control of dental caries
EK and Youssef 2016	Total protein (g/dl) = 0.85 ± 0.71 α-defensin (µg /ml) = 5.43 ± 4.08	Total protein (g/dl) = 0.80 ± 0.74 α-defensin (µg /ml) = 7.16 ± 3.51	*p* = 0.782 *p* = 0.041	Total protein levels similar in both groups α-defensin significantly higher in caries-free subjects	Subjects with caries had low levels of α-defensin & may be used to screen and assess caries susceptibility in children
Monea et al. 2018	Salivary α-amylase Males = 158.18 ± 2.41U/mL Females = 156.83 ± 1.59U/mL	Salivary α-amylase Males = 147.28 ± 2.1U/mL Females = 150.53 ± 2.45 IU/mL	*p* = 0.001 *p* = 0.001	Salivary α-amylase was significantly higher in the caries-active groups	Salivary α-amylase levels could be used to identify high risk individuals as the level of this enzyme increases in caries-active patients
Murugeshappa et al. 2018	Total salivary proteins (mg/mL) = 2.71 sIgA (mcg/mL) = 0.079086	Total salivary proteins (mg/mL) = 1.8 sIgA (mcg/mL) = 0.114286	*p* < 0.01 *p* < 0.01	Mean total salivary protein levels higher in caries-active group Mean sIgA higher in caries-free group	The results show a positive correlation between caries and salivary total proteins and a negative correlation with sIgA
Picco et al. 2017	CA VI conc = 0.4255 ± 0.3835 ng/uL	CA VI conc = 0.8561 ± 0.7141 ng/uL	*p* = 0.0006	CA VI concentration significantly higher in caries-free subjects	Caries is highly affected by the CA VI activity in saliva. The isoenzyme is able to neutralize the acids of oral environment & provide protection against tooth decay
Priya et al. 2013	sIgA = 13.07 (± 1.55) mg/100 ml	sIgA = 11.90 (± 1.58) mg/100 ml	*p* = 0.05	sIgA levels significantly higher in children with caries	Mere quantitation of IgA levels might have no reflection on the functional antibodies involved in caries process
Pyati et al. 2018	Total protein (gm/dl) = 0.41 ± 0.15	Total protein (gm/dl) = 0.34 ± 0.12	*p* = 0.017	Total protein significantly increased in caries-active subjects	Higher total salivary protein levels may indicate caries susceptibility
Ranadheer et al. 2011	sIgA (mg/dl) = 11.760 ± 1.859	sIgA (mg/dl) = 7.585 ± 2.488	*p* = 0.05	Whole s-IgA levels significantly higher in caries-active group with DMFT score ≥ 3	Higher sIgA levels in caries-active subjects may indicate a protective response
Soesilawati et al. 2019	sIgA = 138.334 ± 37.527	sIgA = 545.833 ± 90.298	*p* < 0.001	Total salivary IgA conc significantly higher in low-caries-active group	A negative correlation between sIgA level and caries activity
Stojković et al. 2020	HNP-1 ng/mL = 12.69 ± 5.61 hBD-2 ng/mL = 2.84 ± 1.30 LL-37 ng/mL = 1.74 ± 2.03	HNP-1 ng/mL = 13.02 ± 3.78 hBD-2 ng/mL = 2.84 ± 0.91 LL-37 ng/mL = 1.35 ± 1.30	P = 0.376 *p* = 0.554 *p* = 0.569	The salivary levels of HNP-1, hBD-2, and LL-37 peptides were uniform	Salivary HNP-1, hBD-2, and LL-37 peptides not found to have a predictive value
Vasudevan et al. 2022	CAVI isoenzyme = 1925.54 ± 1398.57 Total protein conc = 1.723 ± 1.943	CAVI isoenzyme = 1444.17 ± 1039.81 Total protein conc = 1.429 ± 1.284	P = 0.135 P = 0.492	CAVI & salivary total protein conc was higher in the caries-active group compared to caries-free group but the difference was not statistically significant	Increased conc of CAVI enzyme in caries‑active group and total protein showed a linear relation with caries activity
Wang et al. 2018	Differentially expressed proteins	Differentially expressed proteins	P < 0.05 Ratio-fold change > 1.2	A total of 244 differentially expressed proteins identified. As compared with NDC group, 62 up-regulated proteins and 28 down-regulated proteins were found in HDC group, while 97 increased proteins & 32 decreased proteins detected in LDC group 53 target proteins with differential expression selected for MRM validation	Key screened proteins: (S100 A9, mucin 7, mucin 5B, statherin, histatin 1, cystatin S, cystatin SN & proline-rich protein 2) are valuable for further validation Salivary proteins with potential anti-cariogenic function can be useful for individualized preventive strategies in future
Yang et al. 2015	Proteinase 3 HDC group = 11.07 ± 7.10 ng/mL LDC group = 12.79 ± 6.19 ng/mL	Proteinase 3 NDC group = 17.82 ± 7.31 ng/mL	*p* < 0.01	The mean PR3 concentration was significantly higher in caries-free group	Salivary proteinase 3 is associated with the severity of caries, with low levels leading to greater severity of caries

CA VI = carbonic anhydrase VI; hBD-2 = human beta-defensin-2; HCR = High caries-risk; HDC = High dental caries; HNP-1 = human alpha defensin; IQR = Interquartile range; LCR = Low caries-risk; LDC = Low dental caries; NDC = No dental caries; NR = Not reported; sIgA = salivary immunoglobulin A

### Total protein content

The association of total protein content with dental caries was investigated by four studies. The total protein content was reported to be positively associated with caries in three studies (Murugeshappa et al. 2018; Pyati et al. 2018; Vasudevan et al. 2022). However, the differences in total salivary protein content between caries-active and caries-free groups were not statistically significant in one study (Vasudevan et al. 2022). One study reported that the total protein content was similar between the two groups (WM and Youssef 2016).

### Salivary glycoproteins

Salivary immunoglobulin A (IgA) was the most commonly investigated glycoprotein in the included studies (*n* = 7). The mean salivary IgA levels were reported to be higher in caries-free group which attributed to their protective role against dental caries (Damle and Doifode 2011; Murugeshappa et al. 2018; Ahmad et al. 2021; Angarita-Díaz et al. 2021). Similarly, salivary IgA levels were reported to be higher in subjects with low-caries activity (Soesilawati et al. 2019). However, salivary IgA levels were observed to be higher in caries-active group in two studies (Ranadheer et al. 2011; Priya et al. 2013).

Increased levels of MUC5B in subjects with low-caries activity and a negative correlation were shown between MUC5B and the number of decayed teeth. On the other hand, the levels of MUC7 were reported to be lower in the low-caries-risk group (Angwaravong et al. 2015).

### Antimicrobial peptides

Salivary levels of cathelicidin LL-37, statherin, and fibronectin were reported to be higher in caries-free groups compared to caries-active group (Angarita-Díaz et al. 2021). Salivary alpha defensin was significantly higher in caries-free subjects (WM and Youssef 2016). However, the salivary levels of antimicrobial peptides HNP-1, hBD-2, and LL-37 were reported to be uniform between caries-active and caries-free groups in another study (Stojković et al. 2020).

### Salivary enzymes

Salivary alpha amylase activity was assessed by two studies: Monea et al., (2018) reported significantly higher levels of the enzyme in caries-active subjects (Monea, Vlad and Stoica 2018). In contrast, another study reported significantly increased levels of salivary amylase in caries-free children (Ahmad et al. 2021). Salivary carbonic anhydrase VI levels were significantly higher in caries-free subjects (Picco et al. 2017)^.^ However, the concentration of carbonic anhydrase VI isoenzyme was reported to be higher in caries-active group in another study, but the difference was not statistically significant (Vasudevan et al. 2022). The mean proteinase 3 concentration was significantly lower in caries-active groups compared to caries-free group (Yang et al. 2015).

### Comprehensive salivary proteomic profile

Two studies undertook a comprehensive evaluation of salivary proteomic profile. Wang et al (2018) identified 244 differentially expressed salivary proteins among children with varying severity of caries. Further analysis highlighted complex protein interactions between various proteins indicating synergistic action of salivary proteins in caries resistance as well as cariogenicity (Wang, et al. 2018). Similarly, Chen et al., (2020) identified 9135 unique peptides and 1662 proteins in 6–8-year-old children. Of these, 258 proteins were differentially expressed between the caries-free and caries-active group (Chen et al. 2020).

### Methodological quality (risk of bias) assessment

The quality assessment for analytical cross-sectional studies was carried out using the JBI critical appraisal tool and is summarized in Table 3. Of the included studies, four were assessed as good quality (Angwaravong et al. 2015; Picco et al. 2017; Pyati et al. 2018; Ahmad et al. 2021), nine presented fair quality (Ranadheer et al. 2011; Priya et al. 2013; Yang et al. 2015; WM and Youssef 2016; Monea, Vlad and Stoica 2018; Murugeshappa et al. 2018; Wang et al. 2018; Chen et al. 2020; Angarita-Díaz et al. 2021), whereas one study was graded as having a poor quality (Damle and Doifode 2011).

**Table 3:** Quality assessment of analytical cross-sectional studies using JBI scale

Primary studies	JBI’s critical appraisal questions								Overall quality score (%)	Quality
	Q1	Q2	Q3	Q4	Q5	Q6	Q7	Q8		
Ahmad et al. 2021	Y	Y	Y	Y	U	U	Y	Y	75%	Good
Angarita-Díaz et al. 2021	Y	Y	Y	Y	U	U	Y	U	62.5%	Fair
Angwaravong et al. 2015	Y	Y	Y	Y	Y	U	Y	Y	87.5%	Good
Chen et al. 2020	Y	Y	Y	Y	U	N	Y	U	62.5%	Fair
Damle and Doifode., 2011	Y	N	Y	U	U	N	Y	U	37.5%	Poor
EK and Youssef., 2016	Y	N	Y	Y	U	N	Y	U	50%	Fair
Monea et al. 2018	Y	Y	Y	Y	U	N	Y	U	62.5%	Fair
Murugeshappa et al. 2018	Y	U	Y	Y	U	U	Y	Y	62.5%	Fair
Picco et al. 2017	Y	Y	Y	U	U	Y	Y	Y	75%	Good
Priya et al. 2013	Y	Y	Y	Y	U	N	Y	N	62.5%	Fair
Pyati et al. 2018	Y	Y	Y	Y	Y	U	Y	Y	87.5%	Good
Ranadheer et al. 2011	Y	N	Y	Y	U	N	Y	Y	62.5%	Fair
Wang et al. 2018	Y	Y	Y	Y	U	U	Y	U	62.5%	Fair
Yang et al. 2015	N	Y	Y	Y	U	U	Y	Y	62.5%	Fair

Y; yes, N; no, U; unclear

For case–control and cohort studies, the Newcastle–Ottawa Scale was used. The results are depicted in Table 4. All the studies were of fair quality (Soesilawati et al. 2019; Stojković et al. 2020; Vasudevan et al. 2022) with a total score ranging from 5 to 6 stars.

**Table 4:** Quality assessment of case–control and cohort studies using Newcastle–Ottawa Scale

Study	Selection (4)	Comparability (2)	Exposure/Outcome (3)	Total score (9)	Quality
Stojković et al. 2020	***	*	**	6	Fair
Soesilawati et al. 2019	***	*	**	6	Fair
Vasudevan et al. 2022	**	*	**	5	Fair

Most studies measured the salivary proteins in a valid and reliable way using objective and standard protein quantification methods. On the contrary, majority of the included studies did not identify confounding factors or not clearly stated strategies to deal with them.

## Discussion

Salivary proteins have gained a growing focus in human diagnostics research in the last two decades partly because salivary samples can be collected using simple and non-invasive methods. Apart from use as biomarkers for risk evaluation and diagnosis of dental caries, the role of salivary proteomics has been investigated for application in the diagnosis of salivary gland disorders (Hu et al. 2010), oral cancer (Mahalingam et al. 2021), and periodontitis (Kaufman and Lamster 2000; Hirtz et al. 2021). In addition, salivary proteomics have been utilized to diagnose systemic disorders, such as cancer, autoimmune diseases, endocrine disorders, and neurological diseases, to name a few (Zhang et al. 2016; Han et al. 2018; Manconi et al. 2018; Stanescu et al. 2018; Sun et al. 2020; MacIejczyk et al. 2021; Mahalingam et al. 2021).

Salivary mucins are glycoproteins, mainly synthesized by the mucus acinar cells of the paired submandibular and sublingual gland as well as minor salivary glands. Salivary mucins are classified as high molecular weight mucins (MG1 or MUC5B) and low molecular weight mucins (MG2 or MUC7). MUC5B provides lubrication and act as a protective barrier. MUC7 plays a key role in agglutination and oral clearance of bacteria (van Nieuw Amerongen et al. 2004). Salivary MUC5B has also been shown to inhibit *S. mutans* attachment and biofilm formation on hydroxyapatite surfaces while MUC7 represents the primary mucin which exerts antimicrobial effect directly and preferentially against S. *mutans*. (Frenkel and Ribbeck 2015). Similarly, s-IgA, another salivary glycoprotein, also exerts an anticaries effect due to inhibition of bacterial adherence, and neutralization of some enzymes and bacterial toxins levels (Fidalgo et al. 2014).

The results of the current review show some obvious contradictions in salivary levels of specific glycoproteins. For example, Angwaravong et al (2015), reported significantly increased MUC5B and decreased MUC7 levels in subjects with low-caries, a finding which is consistent with a previous study (Szkaradkiewicz-Karpińska et al. 2019). However, increased levels of MUC5B in subjects with high caries were reported by another study (Gabryel-Porowska et al. 2014). Similarly conflicting findings are reported for sIgA levels between caries-free and caries-active subjects in the mixed dentition. Five studies in this review reported higher sIgA levels in caries-free subjects (Damle and Doifode 2011; Murugeshappa et al. 2018; Soesilawati et al. 2019; Ahmad et al. 2021; Angarita-Díaz et al. 2021). In contrast, two studies reported that sIgA levels were higher in caries-active subjects (Ranadheer et al. 2011; Priya et al. 2013). Although the total protein content of saliva was reported to be positively associated with caries in two studies included in this review (Murugeshappa et al. 2018; Pyati et al. 2018). Ruan et al (2021) showed that the salivary proteins in caries-free group were statistically greater than those with severe caries during early childhood (Ruan et al. 2021). Such contractions raise questions about the association between expression of caries-protective protein and susceptibility to caries. It is not clear if increased levels of these glycoproteins protect subjects from caries or rise in response to caries or both.

Contradictory findings were also reported for the association of carbonic anhydrase (Picco et al. 2017; Vasudevan et al. 2022) and alpha amylase (Monea, Vlad and Stoica 2018; Ahmad et al. 2021) with caries activity. However, proteins, such as proteinase 3, alpha defensin, lysozyme, and lactoferrin, were only investigated by single studies and it is not possible to corroborate the findings with other studies included in this review. Beyond the studies included in the current review, conflicting results can also be identified in other studies. For example, Vitorino et al., (2006) recorded that statistically significant correlation between the quantity of acidic proline-rich proteins (PRPs), lipocalin, cystatin SN and cystatin in caries-free subjects. (Vitorino et al. 2006) Acidic PRPs were significantly correlated with lower DMFT scores in caries-free group (Vitorino et al. 2006). On the contrary, another study reported that adult subjects with severe caries possessed twice the content of salivary acidic-PRPs (Szkaradkiewicz-Karpinska et al. 2018).

Overall, the results of this review reveal that approximately 62% of salivary proteins show a statistically significant association with caries status of the participants and underscore their potential role as a biomarker of caries. However, the results also highlight the challenges of establishing an association between salivary proteins and susceptibility to dental caries. Given that salivary proteins are endogenous components of saliva, it is not possible to control their expression. Therefore, studies investigating the association of salivary proteins with caries can only rely on the differences in salivary protein levels/expression between caries-free and caries-active subjects. Such limitations preclude the possibility of randomized control clinical trials and studies may need to rely on observational designs only. Moreover, the included studies showed several methodological variations, such as, study design, sample size, caries diagnostic criteria, the specific type of salivary proteins investigated, as well as the characteristics, volume and timing of sample collection. Lack of clarity and variations were also noted in the laboratory tests used for quantification of salivary proteins especially in regard to calibration of reagents and equipment used in different studies. One study did not provide explicit values for protein levels in the results (Angwaravong et al. 2015). The authors were contacted by email to seek clarification and further details, but no response was received. Due to the heterogeneity in methods and results in the included studies, a meta-analysis was not considered to be feasible (Muka et al. 2020).

### Limitations

This systematic review has several limitations which need to be acknowledged. First, a majority of the studies were based on cross-sectional study design which is prone to confounding and temporal ambiguity. The published studies on salivary proteins are based on a single-point measurements of salivary proteins without any longitudinal data. Some studies also show weaknesses in their research design including a small sample size and inadequate consideration of potential confounders. A previous systematic review also identified high risk of bias in published studies and only four studies were found to have a low risk of bias (Martins et al. 2013). Of the 17 studies in this review, the quality of 12 studies was fair. The main issue with the quality of majority of the studies was that confounding factors were not accounted for appropriately. Moreover, the sample size of most studies was relatively small and could limit the generalizability of the results. It is also hard to compare the results of studies that have different research techniques and consequently, their results can be completely different Therefore, the findings of the current review need to be interpreted with a degree of caution.

### Recommendations

The authors recommend future large-scale studies with standardization of sample collection, methodological protocols and laboratory techniques as well as collection of longitudinal data. Further clarity is also required to ascertain if salivary proteins with anti-caries activity rise predictably in caries-free subjects and account for primary caries prevention or whether they show a reactionary rise following increased caries activity. These fundamental questions need to be answered before salivary proteomics are incorporated in the repertoire of caries-risk assessment and diagnostic tools.

## Conclusions

Based on fair-quality studies included in this review and within its limitations, the findings revealed that salivary proteins may be associated with susceptibility to dental caries in mixed dentition. Total salivary proteins are upregulated in caries-active subjects while salivary IgA, antimicrobial peptides and proteinase-3 are increased in caries-free subjects. Further research with studies involving a larger sample size, methodological rigor, and longitudinal follow-up data are recommended to validate the results of published studies and enhance the translational value of salivary proteins in caries-risk assessment.

## Supplementary Information

Below is the link to the electronic supplementary material.

Supplementary file1 (DOCX 51 KB)

## Data Availability

PRISMA checklists, detailed search strategies, and excluded studies (including reasons for exclusion) are included in the appendix: supplementary data.
