# Supplementary material for: Association of salivary proteins with dental caries in children with mixed dentition: a systematic review
Source: Eur Arch Paediatr Dent. 2025 Jan 18;26(4):617–31. doi: 10.1007/s40368-024-00994-4 (PMC12283824; doi:10.1007/s40368-024-00994-4)
Supplement: Supplementary file 1 — Supplementary file1 (DOCX 51 KB) [file 40368_2024_994_MOESM1_ESM.docx]

Title Page:

**Association of salivary proteins with dental caries in children with mixed dentition - A systematic review**

Mahwish Raja^1^, Hani Nazzal^1,2^, Farhan S Cyprian^3^, Manal Matoug- Elwerfelli^1^, Monty Duggal^1^

1. Qatar University Health, College of Dental Medicine, Qatar University, Doha, Qatar

2. Paediatric Dentistry, Hamad Medical Corporation, Doha, Qatar.

3. Qatar University Health, College of Medicine, Qatar University, Doha, Qatar

1. Mahwish Raja (Corresponding author)

Graduate Research Assistant

QU Health, College of Dental Medicine, H12 Building,

Qatar University, Doha, 2713 Qatar

E-mail: [mk2211072@student.qu.edu.qa](mailto:mk2211072@student.qu.edu.qa)

ORCID: [0000-0001-8816-6328](https://orcid.org/0000-0001-8816-6328)

2. Hani Nazzal

Professor of Paediatric Dentistry

QU Health, College of Dental Medicine, Qatar University, Doha, Qatar

Paediatric Dentistry, Hamad Medical Corporation, Doha, Qatar

E-mail: [haninazzal@qu.edu.qa](mailto:haninazzal@qu.edu.qa) [hnazzal@hamad.qa](mailto:hnazzal@hamad.qa)

ORCID: [0000-0002-6220-8873](https://orcid.org/0000-0002-6220-8873)

3. Farhan Sachal Cyprian

Associate Professor of Immunology

QU Health, College of Medicine, Qatar University, Doha, Qatar

E-mail: [fcyprian@qu.edu.qa](mailto:fcyprian@qu.edu.qa)

ORCID: [0000-0001-8162-8673](https://orcid.org/0000-0001-8162-8673)

4. Manal Matoug- Elwerfelli

Assistant Professor of Pre-Clinical Dental Science

QU Health, College of Dental Medicine, Qatar University, Doha, Qatar

E-mail: [melwerfelli@qu.edu.qa](mailto:melwerfelli@qu.edu.qa)

ORCID: [0000-0003-1994-7002](https://orcid.org/0000-0003-1994-7002)

5. Monty Duggal

Professor of Paediatric Dentistry

QU Health ,College of Dental Medicine, Qatar University, Doha, Qatar

E-mail: [mduggal@qu.edu.qa](mailto:mduggal@qu.edu.qa)

ORCID: [0000-0003-3652-5741](https://orcid.org/0000-0003-3652-5741)

Submitted to: European Archives of Paediatric Dentistry

# Supplementary Data: Association of salivary proteins with dental caries in children with mixed dentition

### Table I: PRISMA Checklist

| **Section and Topic** | **Item #** | **Checklist item** | **Location where item is reported** |
| --- | --- | --- | --- |
| **TITLE** | | |  |
| Title | 1 | Identify the report as a systematic review. | Title |
| **ABSTRACT** | | |  |
| Abstract | 2 | See the PRISMA 2020 for Abstracts checklist. | Abstract |
| **INTRODUCTION** | | |  |
| Rationale | 3 | Describe the rationale for the review in the context of existing knowledge. | Introduction |
| Objectives | 4 | Provide an explicit statement of the objective(s) or question(s) the review addresses. | Introduction |
| **METHODS** | | |  |
| Eligibility criteria | 5 | Specify the inclusion and exclusion criteria for the review and how studies were grouped for the syntheses. | Methods |
| Information sources | 6 | Specify all databases, registers, websites, organisations, reference lists and other sources searched or consulted to identify studies. Specify the date when each source was last searched or consulted. | Methods |
| Search strategy | 7 | Present the full search strategies for all databases, registers and websites, including any filters and limits used. | Methods and Supplementary data (Table III) |
| Selection process | 8 | Specify the methods used to decide whether a study met the inclusion criteria of the review, including how many reviewers screened each record and each report retrieved, whether they worked independently, and if applicable, details of automation tools used in the process. | Methods |
| Data collection process | 9 | Specify the methods used to collect data from reports, including how many reviewers collected data from each report, whether they worked independently, any processes for obtaining or confirming data from study investigators, and if applicable, details of automation tools used in the process. | Methods |
| Data items | 10a | List and define all outcomes for which data were sought. Specify whether all results that were compatible with each outcome domain in each study were sought (e.g. for all measures, time points, analyses), and if not, the methods used to decide which results to collect. | Methods |
|  | 10b | List and define all other variables for which data were sought (e.g. participant and intervention characteristics, funding sources). Describe any assumptions made about any missing or unclear information. | Methods |
| Study risk of bias assessment | 11 | Specify the methods used to assess risk of bias in the included studies, including details of the tool(s) used, how many reviewers assessed each study and whether they worked independently, and if applicable, details of automation tools used in the process. | Methods |
| Effect measures | 12 | Specify for each outcome the effect measure(s) (e.g. risk ratio, mean difference) used in the synthesis or presentation of results. | N/A |
| Synthesis methods | 13a | Describe the processes used to decide which studies were eligible for each synthesis (e.g. tabulating the study intervention characteristics and comparing against the planned groups for each synthesis (item #5)). | N/A |
|  | 13b | Describe any methods required to prepare the data for presentation or synthesis, such as handling of missing summary statistics, or data conversions. | Methods |
|  | 13c | Describe any methods used to tabulate or visually display results of individual studies and syntheses. | Methods |
|  | 13d | Describe any methods used to synthesize results and provide a rationale for the choice(s). If meta-analysis was performed, describe the model(s), method(s) to identify the presence and extent of statistical heterogeneity, and software package(s) used. | Methods |
|  | 13e | Describe any methods used to explore possible causes of heterogeneity among study results (e.g. subgroup analysis, meta-regression). | N/A |
|  | 13f | Describe any sensitivity analyses conducted to assess robustness of the synthesized results. | N/A |
| Reporting bias assessment | 14 | Describe any methods used to assess risk of bias due to missing results in a synthesis (arising from reporting biases). | N/A |
| Certainty assessment | 15 | Describe any methods used to assess certainty (or confidence) in the body of evidence for an outcome. | Methods |
| **RESULTS** | | |  |
| Study selection | 16a | Describe the results of the search and selection process, from the number of records identified in the search to the number of studies included in the review, ideally using a flow diagram. | Figure 1 |
|  | 16b | Cite studies that might appear to meet the inclusion criteria, but which were excluded, and explain why they were excluded. | Supplementary data Table IV |
| Study characteristics | 17 | Cite each included study and present its characteristics. | Table 1 and Table 2 |
| Risk of bias in studies | 18 | Present assessments of risk of bias for each included study. | Results Tables 3 and 4 |
| Results of individual studies | 19 | For all outcomes, present, for each study: (a) summary statistics for each group (where appropriate) and (b) an effect estimates and its precision (e.g. confidence/credible interval), ideally using structured tables or plots. | Results |
| Results of syntheses | 20a | For each synthesis, briefly summarise the characteristics and risk of bias among contributing studies. | Results |
|  | 20b | Present results of all statistical syntheses conducted. If meta-analysis was done, present for each the summary estimate and its precision (e.g. confidence/credible interval) and measures of statistical heterogeneity. If comparing groups, describe the direction of the effect. | Results |
|  | 20c | Present results of all investigations of possible causes of heterogeneity among study results. | N/A |
|  | 20d | Present results of all sensitivity analyses conducted to assess the robustness of the synthesized results. | N/A |
| Reporting biases | 21 | Present assessments of risk of bias due to missing results (arising from reporting biases) for each synthesis assessed. | N/A |
| Certainty of evidence | 22 | Present assessments of certainty (or confidence) in the body of evidence for each outcome assessed. | Results |
| **DISCUSSION** | | |  |
| Discussion | 23a | Provide a general interpretation of the results in the context of other evidence. | Discussion |
|  | 23b | Discuss any limitations of the evidence included in the review. | Discussion |
|  | 23c | Discuss any limitations of the review processes used. | Discussion |
|  | 23d | Discuss implications of the results for practice, policy, and future research. | Discussion |
| **OTHER INFORMATION** | | |  |
| Registration and protocol | 24a | Provide registration information for the review, including register name and registration number, or state that the review was not registered. | Methods |
|  | 24b | Indicate where the review protocol can be accessed, or state that a protocol was not prepared. | Methods |
|  | 24c | Describe and explain any amendments to information provided at registration or in the protocol. | N/A |
| Support | 25 | Describe sources of financial or non-financial support for the review, and the role of the funders or sponsors in the review. | Declarations |
| Competing interests | 26 | Declare any competing interests of review authors. | Declarations |
| Availability of data, code and other materials | 27 | Report which of the following are publicly available and where they can be found template data collection forms; data extracted from included studies; data used for all analyses; analytic code; any other materials used in the review. | Supplementary data |

*From:*  Page MJ, McKenzie JE, Bossuyt PM, Boutron I, Hoffmann TC, Mulrow CD, et al. The PRISMA 2020 statement: an updated guideline for reporting systematic reviews. BMJ 2021;372:n71. doi: 10.1136/bmj.n71

For more information, visit: <http://www.prisma-statement.org/>

**Table II: PRISMA Checklist for Abstracts**

| **Section and Topic** | **Item #** | **Checklist item** | **Reported (Yes/No)** |
| --- | --- | --- | --- |
| **TITLE** | | |  |
| Title | 1 | Identify the report as a systematic review. | Y |
| **BACKGROUND** | | |  |
| Objectives | 2 | Provide an explicit statement of the main objective(s) or question(s) the review addresses. | Y |
| **METHODS** | | |  |
| Eligibility criteria | 3 | Specify the inclusion and exclusion criteria for the review. | Y |
| Information sources | 4 | Specify the information sources (e.g. databases, registers) used to identify studies and the date when each was last searched. | Y |
| Risk of bias | 5 | Specify the methods used to assess risk of bias in the included studies. | Y |
| Synthesis of results | 6 | Specify the methods used to present and synthesise results. | Y |
| **RESULTS** | | |  |
| Included studies | 7 | Give the total number of included studies and participants and summarise relevant characteristics of studies. | Y |
| Synthesis of results | 8 | Present results for main outcomes, preferably indicating the number of included studies and participants for each. If meta-analysis was done, report the summary estimate and confidence/credible interval. If comparing groups, indicate the direction of the effect (i.e. which group is favoured). | Y |
| **DISCUSSION** | | |  |
| Limitations of evidence | 9 | Provide a brief summary of the limitations of the evidence included in the review (e.g. study risk of bias, inconsistency and imprecision). | Y |
| Interpretation | 10 | Provide a general interpretation of the results and important implications. | Y |
| **OTHER** | | |  |
| Funding | 11 | Specify the primary source of funding for the review. | Y |
| Registration | 12 | Provide the register name and registration number. | Y |

### Table III: Search strategy

| **PubMed** | | |
| --- | --- | --- |
| *Search* | *Query* | *Items found* |
| #1 | ((((((((((((((((((((("saliva proteins") OR ("saliva peptides")) OR ("saliva proteome")) OR ("salivary proteins")) OR ("salivary peptides")) OR ("Mucin-5B"[Mesh])) OR ("Salivary Proline-Rich Proteins"[Mesh])) OR ("Salivary alpha-Amylases"[Mesh])) OR ("Histatins"[Mesh])) OR ("Salivary Cystatins"[Mesh])) OR ("salivary protein biomarkers")) OR ("salivary proteomic profile")) OR ("total salivary proteins")) OR ("salivary mucins")) OR ("salivary IgA")) OR ("salivary statherin")) OR ("salivary defensins")) OR (salivary cathelicidins)) OR (salivary human lysozyme)) OR ("salivary lactoferrin")) OR ("salivary glycoproteins")) OR (salivary proteinase 3) | 8,666 |
| #2 | (((((((("Dental Caries"[Mesh]) OR ("Dental Caries Susceptibility"[Mesh])) OR ("caries")) OR ("carious lesion")) OR ("tooth demineralisation")) OR ("dental decay")) OR ("tooth cavities")) OR ("white spot lesions")) OR ("tooth decay") | 71,493 |
| #3 | #1 **AND** #2 | 557 |
|  | (((((((((((((((((((((("saliva proteins") OR ("saliva peptides")) OR ("saliva proteome")) OR ("salivary proteins")) OR ("salivary peptides")) OR ("Mucin-5B"[Mesh])) OR ("Salivary Proline-Rich Proteins"[Mesh])) OR ("Salivary alpha-Amylases"[Mesh])) OR ("Histatins"[Mesh])) OR ("Salivary Cystatins"[Mesh])) OR ("salivary protein biomarkers")) OR ("salivary proteomic profile")) OR ("total salivary proteins")) OR ("salivary mucins")) OR ("salivary IgA")) OR ("salivary statherin")) OR ("salivary defensins")) OR (salivary cathelicidins)) OR (salivary human lysozyme)) OR ("salivary lactoferrin")) OR ("salivary glycoproteins")) OR (salivary proteinase 3)) **AND** ((((((((("Dental Caries"[Mesh]) OR ("Dental Caries Susceptibility"[Mesh])) OR ("caries")) OR ("carious lesion")) OR ("tooth demineralisation")) OR ("dental decay")) OR ("tooth cavities")) OR ("white spot lesions")) OR ("tooth decay")) |  |
| #4 | Filters | 384 |
|  | (((((((((((((((((((((("saliva proteins") OR ("saliva peptides")) OR ("saliva proteome")) OR ("salivary proteins")) OR ("salivary peptides")) OR ("Mucin-5B"[Mesh])) OR ("Salivary Proline-Rich Proteins"[Mesh])) OR ("Salivary alpha-Amylases"[Mesh])) OR ("Histatins"[Mesh])) OR ("Salivary Cystatins"[Mesh])) OR ("salivary protein biomarkers")) OR ("salivary proteomic profile")) OR ("total salivary proteins")) OR ("salivary mucins")) OR ("salivary IgA")) OR ("salivary statherin")) OR ("salivary defensins")) OR (salivary cathelicidins)) OR (salivary human lysozyme)) OR ("salivary lactoferrin")) OR ("salivary glycoproteins")) OR (salivary proteinase 3)) **AND** ((((((((("Dental Caries"[Mesh]) OR ("Dental Caries Susceptibility"[Mesh])) OR ("caries")) OR ("carious lesion")) OR ("tooth demineralisation")) OR ("dental decay")) OR ("tooth cavities")) OR ("white spot lesions")) OR ("tooth decay")) AND ((humans[Filter]) AND (english[Filter])) |  |
| **Scopus** | | |
| *Search* | *Query* | *Items found* |
| #1 | TITLE-ABS-KEY( "saliva proteins" OR "saliva peptides" OR "saliva proteome" OR "salivary proteins" OR "salivary peptides" OR "Mucin-5B" OR "Salivary Proline-Rich Proteins" OR "Salivary alpha-Amylases" OR "Histatins" OR "Salivary Cystatins" OR "salivary protein biomarkers" OR "salivary proteomic profile" OR "total salivary proteins" OR "salivary mucins" OR "salivary IgA" OR "salivary statherin" OR "salivary defensins" OR "salivary cathelicidins" OR "salivary human lysozyme" OR "salivary lactoferrin" OR "salivary glycoproteins" OR "salivary proteinase 3") | 11,804 |
| #2 | TITLE-ABS-KEY ("dental Caries" OR "dental caries susceptibility" OR "carious lesion" OR "tooth demineralization" OR "dental decay" OR "tooth cavities" OR "caries" OR "white spot lesions" OR "tooth decay") | 88,792 |
| #3 | #1 **AND** #2 | 658 |
|  | (TITLE-ABS-KEY( "saliva proteins" OR "saliva peptides" OR "saliva proteome" OR "salivary proteins" OR "salivary peptides" OR "Mucin-5B" OR "Salivary Proline-Rich Proteins" OR "Salivary alpha-Amylases" OR "Histatins" OR "Salivary Cystatins" OR "salivary protein biomarkers" OR "salivary proteomic profile" OR "total salivary proteins" OR "salivary mucins" OR "salivary IgA" OR "salivary statherin" OR "salivary defensins" OR "salivary cathelicidins" OR "salivary human lysozyme" OR "salivary lactoferrin" OR "salivary glycoproteins" OR "salivary proteinase 3")) **AND** (TITLE-ABS-KEY ("dental Caries" OR "dental caries susceptibility" OR "carious lesion" OR "tooth demineralization" OR "dental decay" OR "tooth cavities" OR "caries" OR "white spot lesions" OR "tooth decay")) |  |
| #4 | Filters | 435 |
|  | (TITLE-ABS-KEY( "saliva proteins" OR "saliva peptides" OR "saliva proteome" OR "salivary proteins" OR "salivary peptides" OR "Mucin-5B" OR "Salivary Proline-Rich Proteins" OR "Salivary alpha-Amylases" OR "Histatins" OR "Salivary Cystatins" OR "salivary protein biomarkers" OR "salivary proteomic profile" OR "total salivary proteins" OR "salivary mucins" OR "salivary IgA" OR "salivary statherin" OR "salivary defensins" OR "salivary cathelicidins" OR "salivary human lysozyme" OR "salivary lactoferrin" OR "salivary glycoproteins" OR "salivary proteinase 3")) **AND** (TITLE-ABS-KEY ("dental Caries" OR "dental caries susceptibility" OR "carious lesion" OR "tooth demineralization" OR "dental decay" OR "tooth cavities" OR "caries" OR "white spot lesions" OR "tooth decay")) AND ( LIMIT-TO ( EXACTKEYWORD,"Human" ) ) AND ( LIMIT-TO ( LANGUAGE,"English" ) ) |  |
| **Embase** | | |
| *Search* | *Query* | *Items found* |
| #1 | 'saliva protein'/exp OR 'mucin 5b'/exp OR 'proline rich protein'/exp OR 'alpha amylase saliva isoenzyme'/exp OR 'histatin'/exp OR 'cystatin s'/exp OR 'salivary peptides' OR 'salivary protein biomarkers' OR 'salivary proteomic profile' OR 'total salivary proteins' OR 'salivary mucins' OR 'salivary iga' OR 'salivary statherin' OR 'salivary defensins' OR 'salivary cathelicidins' OR 'salivary human lysozyme' OR 'salivary lactoferrin’ OR 'salivary glycoproteins' OR 'salivary proteinase 3' | 10,574 |
| #2 | 'dental caries'/exp OR 'tooth demineralization'/exp OR 'dental caries susceptibility' OR 'carious lesion' OR 'dental decay' OR 'tooth cavities' OR 'caries' OR 'white spot lesions' OR 'tooth decay' | 79,754 |
| #3 | #1 **AND** #2 | 557 |
|  | ('saliva protein'/exp OR 'mucin 5b'/exp OR 'proline rich protein'/exp OR 'alpha amylase saliva isoenzyme'/exp OR 'histatin'/exp OR 'cystatin s'/exp OR 'salivary peptides' OR 'salivary protein biomarkers' OR 'salivary proteomic profile' OR 'total salivary proteins' OR 'salivary mucins' OR 'salivary iga' OR 'salivary statherin' OR 'salivary defensins' OR 'salivary cathelicidins' OR 'salivary human lysozyme' OR 'salivary lactoferrin' OR 'salivary glycoproteins' OR 'salivary proteinase 3') **AND** ('dental caries'/exp OR 'tooth demineralization'/exp OR 'dental caries susceptibility' OR 'carious lesion' OR 'dental decay' OR 'tooth cavities' OR 'caries' OR 'white spot lesions' OR 'tooth decay') |  |
| #4 | Filters | 405 |
|  | ('saliva protein'/exp OR 'mucin 5b'/exp OR 'proline rich protein'/exp OR 'alpha amylase saliva isoenzyme'/exp OR 'histatin'/exp OR 'cystatin s'/exp OR 'salivary peptides' OR 'salivary protein biomarkers' OR 'salivary proteomic profile' OR 'total salivary proteins' OR 'salivary mucins' OR 'salivary iga' OR 'salivary statherin' OR 'salivary defensins' OR 'salivary cathelicidins' OR 'salivary human lysozyme' OR 'salivary lactoferrin' OR 'salivary glycoproteins' OR 'salivary proteinase 3') **AND** ('dental caries'/exp OR 'tooth demineralization'/exp OR 'dental caries susceptibility' OR 'carious lesion' OR 'dental decay' OR 'tooth cavities' OR 'caries' OR 'white spot lesions' OR 'tooth decay') AND [humans]/lim AND [english]/lim |  |
|  | **Google Scholar** |  |
| #2 | “saliva proteins” AND “dental caries”  <https://scholar.google.com/scholar?hl=en&as_sdt=0%2C5&as_vis=1&q=%E2%80%9Csaliva+proteins%E2%80%9D+AND+%E2%80%9Cdental+caries%E2%80%9D&btnG=> | 811 |

### Table IV: Studies excluded at full text, including the reasons for exclusion.

| S.No | Author/Year | Title of Study | Reason For Exclusion |
| --- | --- | --- | --- |
|  | Al Amoudi et al 2007 | A comparative study of the secretory IgA immunoglobulins (sIgA) in mothers and children with SECC versus a caries free group children and their mothers | Study population does not match inclusion criteria |
|  | Alaçam et al 1986 | The relationship between the caries activity test (Snyder) and salivary IgA level | Study population does not match inclusion criteria |
|  | Anderson 1981 | Salivary proteins and dental caries: genetic considerations | Genetic polymorphism |
|  | Ayad et al 2000 | The association of basic proline-rich peptides from human parotid gland secretions with caries experience | Study population does not match inclusion criteria |
|  | Bachtiar et al 2018 | Analysis of Salivary Protein Profiles and its Viscosity in Early Childhood Caries (A Cross-Sectional Study) | Study population does not match inclusion criteria |
|  | Banderas-Tarabay et al 2002 | Electrophoretic analysis of whole saliva and prevalence of dental caries. A study in Mexican dental students | Study population does not match inclusion criteria |
|  | Bardow et al 2005 | Effect of saliva composition on experimental root caries | Study population does not match inclusion criteria |
|  | Beeley 1991 | Clinical applications of electrophoresis of human salivary proteins | Review |
|  | Bhatia et al 1986 | Naturally occurring s-IgA saliva of adults and children--correlation with dental caries activity | No abstract available |
|  | Bolton 1981 | Naturally occurring IgA antibodies to glycerol-teichoic acid in human saliva. Correlation with caries activity | Salivary IgA levels of TA specificity has been reported. |
|  | Bolton et al 1982 | Evaluation of salivary IgA antibodies to cariogenic microorganisms in children. Correlation with dental caries activity | Study population does not match inclusion criteria |
|  | Bratthall et al 1997 | Immunoglobulin A reaction to oral streptococci in saliva of subjects with different combinations of caries and levels of mutans streptococci | Salivary antibody response to S. Mutans were observed.  Study population does not match inclusion criteria |
|  | Chia et al 1997 | Antigenicity of a synthetic peptide from glucosyltransferases of Streptococcus mutans in humans | Association of dental caries and antibody levels to Gtfs by using synthetic peptides and purified S. mutans Gtfs were observed. |
|  | Davidopoulou et al 2012 | Salivary concentration of the antimicrobial peptide LL-37 in children | Study population does not match inclusion criteria |
|  | Dodds et al 1997 | Parotid saliva protein profiles in caries-free and caries-active adults. | Study population does not match inclusion criteria |
|  | Everhart et al1977 | Evaluation of Dental Caries Experience and Salivary IgA in Children Ages 3–7. | Study population does not match inclusion criteria |
|  | Farias et al 2003 | Salivary antibodies, amylase and protein from children with early childhood caries | Study population does not match inclusion criteria |
|  | Frenkel et al 2015 | Salivary mucins protect surfaces from colonization by cariogenic bacteria | Different aim |
|  | Furlan et al2012 | Salivary cortisol, alpha-amylase and heart rate variation in response to dental treatment in children | Study population does not match inclusion criteria |
|  | Gregory et al 1985 | Prevention of Streptococcus mutans colonization by salivary IgA antibodies | Study design does not match inclusion criteria. |
|  | Hertel et al 2022 | Mucins 5b and 7 and secretory IgA in the oral acquired pellicle of children with caries and caries-free children | Study population does not match inclusion criteria |
|  | Jha et al 2022 | Role of Salivary Physicochemical and Peptide Levels in Dental Caries among Children: An Original Research | Study population does not match inclusion criteria |
|  | Jonasson et al 2007 | Innate immunity glycoprotein gp-340 variants may modulate human susceptibility to dental caries. | Association of the gp-340 I to III polymorphisms with caries experience and adhesion of S. mutans has been observed |
|  | Jurczak et al 2015 | A study on β-defensin-2 and histatin-5 as a diagnostic marker of early childhood caries progression. | Study population does not match inclusion criteria. |
|  | Kargül et al 1994 | Salivary protein and some inorganic element levels in healthy children and their relationship to caries | Full text not retrievable |
|  | Kedjarune et al 1997 | Flow Rate and Composition of Whole Saliva in Children from Rural and Urban Thailand with Different Caries Prevalence and Dietary Intake | Study population does not match inclusion criteria |
|  | Khan et al 2021 | Differentially Expressed Salivary Proteins in Dental Caries Patients | Study population does not match inclusion criteria |
|  | Kirstilä et al 1998 | Longitudinal analysis of the association of human salivary antimicrobial agents with caries increment and cariogenic micro-organisms: a two-year cohort study | Study population does not match inclusion criteria |
|  | Kivelä et al 1999 | A Low Concentration of Carbonic Anhydrase Isoenzyme VI in Whole Saliva Is Associated with Caries Prevalence | Study population does not match inclusion criteria |
|  | Kugler et al 1996 | Excavation of caries lesions induces transient decrease of total salivary immunoglobulin A concentration. | This study investigated how excavation of caries lesions affects total salivary IgA concentration. (Different aim) |
|  | Kulhavá et al 2018 | Differences of Saliva Composition in Relation to Tooth Decay and Gender | Study population does not match inclusion criteria |
|  | Lagerlöf, et al 1994 | Caries-protective factors in saliva | Review |
|  | Lertsirivorakul et al 2015 | Salivary Lysozyme in Relation to Dental Caries among Thai Preschoolers | Study population does not match inclusion criteria |
|  | Mahjoub et al 2014 | Comparison of total antioxidant capacity in saliva of children with severe early childhood caries and caries-free children | Total antioxidant capacity (TAC) levels are compared |
|  | Malberti et al 2004 | Oral health and salivary factors in rural school children | Study population does not match inclusion criteria |
|  | Mendel et al 1983 | Quantitation of human salivary acidic proline-rich proteins in oral diseases | Study population does not match inclusion criteria |
|  | Nahas et al 2020 | Salivary Immunoglobulin A and Streptococcus mutans Levels among Lebanese Preschool Children with Early Childhood Caries | Study population does not match inclusion criteria |
|  | Omar et al2012 | Glucosyltransferase B, immunoglobulin a, and caries experience among a group of Egyptian preschool children | Study population does not match inclusion criteria |
|  | Piotrowski et al 1992 | Expression of salivary mucin bacterial aggregating activity: difference with caries | Different aim |
|  | Rose et al 1994 | IgA antibodies to Streptococcus mutans in caries-resistant and -susceptible children | Could not retrieve full text |
|  | Rudney et al 2009 | Potential biomarkers of human salivary function: a modified proteomic approach | Different aim |
|  | Scannapieco et al 1993 | Salivary alpha-amylase: role in dental plaque and caries formation | Review |
|  | Shifa et al 2008 | Quantitative assessment of IgA levels in the unstimulated whole saliva of caries-free and caries-active children | Study population does not match inclusion criteria |
|  | Shomers et al1982 | Properties of cysteine-containing phosphoproteins from human submandibular-sublingual saliva | Study population does not match inclusion criteria |
|  | Siqueira et al 2010 | Evidence of intact histatins in the in vivo acquired enamel pellicle | The study demonstrated the presence of intact histatins in vivo in the AEP. |
|  | Slomiany et al 1993 | Differential expression of salivary mucin bacterial aggregating activity with caries status | The low and high mol. wt mucin forms were assessed for their bacterial aggregating potential towards S. mutans and S. sanguis. |
|  | Slomiany et al1987 | Buoyant Density and Viscosity Behavior of Salivary Mucin from Individuals with Different Caries Status | Study population does not match inclusion criteria |
|  | Tao et al 2005 | Salivary antimicrobial peptide expression and dental caries experience in children | Study population does not match inclusion criteria |
|  | Tenovuo, et al 1987 | Antimicrobial factors in saliva: ontogeny and relation to oral health | Study population does not match inclusion criteria |
|  | Tenovuo, et al 1987 | Serum and Salivary Antibodies against Streptococcus mutans in Young Children with and without Detectable Oral S. mutans | Avidity of specific antibodies against S. mutans serotype c in serum and saliva were observed |
|  | Tenovuo1991 | Antibody responses to mutans streptococci in children | Review |
|  | Tulunoglu et al 2005 | Total antioxidant levels of saliva in children related to caries, age, and gender. | Study population does not match inclusion criteria |
|  | Twetman et al 1981 | Lysozyme and salivary immunoglobulin A in caries-free and caries-susceptible pre-school children | Wrong Study Population |
|  | Vacaru et al 2022 | Salivary Enzymatic Activity and Carious Experience in Children: A Cross-Sectional Study. | Study population does not match inclusion criteria |
|  | Vitorino et al 2005 | The role of salivary peptides in dental caries | Salivary peptides expression levels and adsorption to enamel surface have been evaluated. Study population does not match inclusion criteria |
|  | Vitorino et al 2006 | Salivary clinical data and dental caries susceptibility: is there a relationship? | Full text not retrievable |
|  | Vitorino et al 2006 | Two-dimensional electrophoresis study of in vitro pellicle formation and dental caries susceptibility | Study evaluated the influence of salivary protein composition on in vitro dental pellicle formation. |
|  | Vukosavljevic et al 2011 | Saliva proteins as predictors and controls of oral health | Commentary |
|  | Zakhary et al 2007 | Acidic proline-rich protein Db and caries in young children. | Acidic PRP alleles of the PRH1 locus (Db) using genomic DNA, to determine its association with caries |
|  | Zehetbauer et al 2009 | Resemblance of salivary protein profiles between children with early childhood caries and caries-free controls | Study population does not match inclusion criteria. |
|  |  |  |  |
